# H_2_O_2_-Induced Oxidative Stress Affects SO_4_^=^ Transport in Human Erythrocytes

**DOI:** 10.1371/journal.pone.0146485

**Published:** 2016-01-08

**Authors:** Rossana Morabito, Orazio Romano, Giuseppa La Spada, Angela Marino

**Affiliations:** 1 Department of Human and Social Sciences, University of Messina, Messina, Italy; 2 BromaTech S.r.l., Giarre, Italy; 3 Department of Biological and Environmental Sciences, University of Messina, Messina, Italy; Institut national de la santé et de la recherche médicale - Institut Cochin, FRANCE

## Abstract

The aim of the present investigation was to verify the effect of H_2_O_2_-induced oxidative stress on SO_4_^=^ uptake through Band 3 protein, responsible for Cl^-^/HCO_3_^-^ as well as for cell membrane deformability, due to its cross link with cytoskeletal proteins. The role of cytoplasmic proteins binding to Band 3 protein has been also considered by assaying H_2_O_2_ effects on hemoglobin-free resealed ghosts of erythrocytes. Oxidative conditions were induced by 30 min exposure of human erythrocytes to different H_2_O_2_ concentrations (10 to 300 μM), with or without GSH (glutathione, 2 mM) or curcumin (10 μM), compounds with proved antioxidant properties. Since SO_4_^=^ influx through Band 3 protein is slower and better controllable than Cl^-^ or HCO_3_^-^ exchange, the rate constant for SO_4_^=^ uptake was measured to prove anion transport efficiency, while MDA (malondialdehyde) levels and –SH groups were estimated to quantify the effect of oxidative stress. H_2_O_2_ induced a significant decrease in rate constant for SO_4_^=^ uptake at both 100 and 300 μM H_2_O_2_. This reduction, observed in erythrocytes but not in resealed ghosts and associated to increase in neither MDA levels nor in –SH groups, was impaired by both curcumin and GSH, whereas only curcumin effectively restored H_2_O_2_-induced changes in erythrocytes shape. Our results show that: i) 30 min exposure to 300 μM H_2_O_2_ reduced SO_4_^=^ uptake in human erythrocytes; ii) oxidative damage was revealed by the reduction in rate constant for SO_4_^=^ uptake, but not by MDA or –SH groups levels; iii) the damage was produced *via* cytoplasmic components which cross link with Band 3 protein; iv) the natural antioxidant curcumin may be useful in protecting erythrocytes from oxidative injury; v) SO_4_^=^ uptake through Band 3 protein may be reasonably suggested as a tool to monitor erythrocytes function under oxidative conditions possibly deriving from alcohol consumption, use of drugs, radiographic contrast media administration, hyperglicemia or neurodegenerative diseases.

## Introduction

The erythrocyte membrane consists of a phospholipid bilayer with integral proteins associated to cytoskeleton through a proteins network underlying the cytoplasmatic side of the membrane [1]. As frequently exposed to oxidative events, it represents a model to study the effect of oxidative stress. In this regard, H_2_O_2_, Cu^2+^-ascorbic acid, Fe^2+^-ascorbic acid, azocompounds, known to be oxidant substances, and their effects, such as methemoglobin production, lipid peroxidation and spectrin-hemoglobin (Hb) complexes, have been long investigated [2,3]. Membrane rigidity induced by oxidative stress has been also observed, mainly due to a reduction in mobility of the proteins embedded in the phospholipidic bilayer [4].

One of the most studied integral membrane proteins is Band 3 protein, particularly abundant in erythrocytes [5]. It is a Cl^-^/HCO_3_^-^ exchanger responsible for gas exchange, ion balance across cell membrane, osmotic and mechanical properties of the erythrocyte, such as anchoring motifs for the glycolytic enzymes and cell shape maintaining [6]. These functions are mediated by two domains, a membrane domain for anion exchange and a cytoplasmic domain which mainly contributes to the protein–protein interactions, by coupling the lipid bilayer to the underlying cytoskeleton, through cysteine -SH groups [7]. It has been demonstrated that deficiencies in Band 3 protein are responsible for a reduced cohesion between the lipid bilayer and cytoskeleton, with consequent shape changes, leading to spherocytosis [8]. Band 3 protein interaction with intracellular components, namely hemoglobin [9,10,11], have been also described.

In an attempt to better clarify the response of Band 3 protein to external stressors, in line with what previously demonstrated [12,13], and since the study of H_2_O_2_ effects on human erythrocytes has been rather limited to a morphological level [2,14], the present investigation aims to demonstrate whether and how Band 3 protein function, monitored through SO_4_^=^ uptake measurement, is affected by H_2_O_2_-induced oxidative stress in human erythrocytes, at concentrations below 1 mM considered physiological and nontoxic [15].

To accomplish this goal, the rate constant for SO_4_^=^ transport, slower and better controllable than Cl^-^ or HCO_3_^-^ uptake and, hence, more easily estimated [16,17], has been measured by a turbidimetric method [12], to monitor Band 3 protein efficiency. Oxidative damage, induced by exposure of erythrocytes to H_2_O_2_ (10 to 300 μM), has been assessed by MDA (Malondialdehyde) assay and –SH group detection and verified after treatment with antioxidant compounds, such as GSH (Glutathione) and curcumin (Curcumin (1,7-bis(4-hydroxy-3-methoxyphenyl)-1E,6E-heptadiene-3,5-dione), a yellow hydrophobic pigment deriving from the rhizome (turmeric) of *Curcuma longa* herb, frequently used in foods and known to have antioxidant properties [18]. The use of curcumin in therapeutics is also reported [19,20]. Furthermore, the involvement of intracellular content in H_2_O_2_–induced alterations of SO_4_^=^ transport has been evaluated on hemoglobin-free resealed ghosts of erythrocytes [13,21].

## Materials and Methods

### Ethics statement

The study was conducted after informed written consent of healthy volunteers during routine medical purposes. The informed consent covered the use of blood for research scopes. P. Romano, MD, from Clinical Pathology of Ospedale Maggiore Modica (Ragusa, Italy) collected and anonymized blood samples, according to the local ethics committee guidelines(S1 text). Samples were collected during 2012 and handled as described in our previous investigations [12,22,23,24].

### Erythrocytes preparation

Blood was collected in heparinized tubes, washed in an isotonic solution (145 mM NaCl, 20 mM HEPES (4-(2-hydroxyethyl)-1-piperazineethanesulfonic acid), pH 7.4, osmotic pressure 300 mOsm), henceforth defined as isotonic medium, and centrifuged three times (Thermoscientific, 1200 g, 5 min) to remove plasma and buffy coat. Erythrocytes were then suspended to either 3% hematocrit (for SO_4_^=^ uptake measurement and for obtaining hemoglobin-free resealed ghosts), while were suspended to 10% hematocrit for MDA assay and –SH groups estimation. Blood samples with normal hemoglobin were used.

### SO_4_^=^ uptake measurement in intact erythrocytes

SO_4_^=^ uptake is used to study Band 3 protein functionality in erythrocytes washed and incubated with Cl^-^-free buffer [16]. In these conditions, the intracellular Cl^-^ content is reduced, while the influx of SO_4_^=^ increases due to the absence of competing extracellular Cl^-^ [25].

To measure SO_4_^=^ uptake, erythrocytes were suspended to 3% hematocrit in 35 ml isotonic medium, henceforth defined as SO_4_^=^ medium, containing 118 mM Na_2_SO_4_, 20 mM HEPES, 15 mM glucose, pH 7.4, osmotic pressure 300 mOsm. At specified time intervals (5-15-30-45-60-90-120 min), 5 ml samples of erythrocytes suspension were removed and added to a test tube containing 10 μM DIDS (4,4’-diisothiocyanato-stilbene-2,2’-disulfonate) stopping medium and kept under ice. DIDS blocks Band 3 protein by irreversibly and specifically binding its extracellular moiety [26]. Erythrocytes were observed under a light microscope (Leica DMLS, 400x) at 5 min and 90 min of incubation in SO_4_^=^ medium and an estimation of damaged erythrocytes was provided by cell count *per* microscopic field (400x). Percentage of deformed erythrocytes, in both control and experimental conditions, was expressed as a mean value deriving from cell count *per* 5 fields.

After the last sample withdrawal, erythrocytes were washed three times in cold isotonic medium (4°C, 1200 g, 5 min) to remove SO_4_^=^ from the external medium. Cells were then hemolysed by 1 ml distilled water and proteins were hydrolysed by 4% v/v Perchloric acid. Cell membranes were discarded by centrifugation (4°C, 2700 g, 10 min) and SO_4_^=^ in the supernatant was precipitated by sequentially adding 1 ml glycerol and distilled water solution (1:1), 1 ml 4 M NaCl plus HCl (hydrochloric acid 37% v/v) solution (12:1) and 500 μl 1.24 M BaCl_2_•2H_2_O to 500 μl supernatant from each sample. Levels of SO_4_^=^ were spectrophotometrically measured at 425 nm wavelength (Beckman DU 640). Using a calibrated standard curve obtained by precipitating known SO_4_^=^ concentrations, the absorption was converted to mM of intracellular SO_4_^=^, necessary to calculate the rate constant in min^-1^, derived from the following equation: C_t_ = C_∞_ (1-e^-rt^) + C_0_, where C_t_, C_∞_ and C_0_ represent the intracellular SO_4_^=^ concentrations measured at time, *t 0* and ∞ respectively; *e* indicates the Neper number (2.7182818), *r* is a constant accounting for specific velocity of the process and *t* is time at which intracellular SO_4_^=^ concentration is measured [27]. SO_4_^=^ uptake was measured as [SO_4_^=^] L cells x10^-2^.

### Preparation of hemoglobin-free ghosts of erythrocytes and SO_4_^=^ uptake measurement

Pink resealed ghosts of human erythrocytes were prepared as reported elsewhere [13], with slight modifications. In detail, erythrocytes, after washing, were re-suspended at 3% hematocrit in 35 ml hyposmotic medium (2.5 mM NaH_2_PO_4_, 5 mM HEPES, pH 7.4). After 10 min stirring at 0°C, hemoglobin and intracellular content were eliminated by repeated centrifugations (Beckman J2-21, 4°C, 17000 g, 20 min). After the last centrifugation, the supernatant was removed and replaced with 35 ml isotonic resealing medium (145 mM NaCl, 20 mM HEPES, 5 mM glucose, pH 7.4), pre-heated at 37°C. Membranes were incubated at 37°C for 45 min, to allow resealing. Then, pink resealed ghosts, containing about 10% of the original hemoglobin, were used for SO_4_^=^ uptake measurement, following the protocol above described for intact erythrocytes.

### SO_4_^=^ uptake measurement after H_2_O_2_ treatment

After rate constant for SO_4_^=^ transport assessment in control conditions the following experimental protocols were used:

i) intact erythrocytes, after washing, were diluted to 3% hematocrit in isotonic medium plus H_2_O_2_ at different concentrations (10-100-300 μM respectively). After 30 min incubation at 25°C, samples were centrifuged to remove the supernatant and erythrocytes re-suspended to 3% hematocrit in SO_4_^=^ medium. SO_4_^=^ uptake was measured following what described for control conditions. Erythrocytes were observed under a light microscope at 5 min and 90 min of incubation in SO_4_^=^ medium and damaged cells counted *per* microscopic field at the end of incubation.

ii) intact erythrocytes, after washing, were diluted to 3% hematocrit in isotonic medium containing either 2 mM GSH or, alternatively, 10 μM curcumin [28], as antioxidant compounds. Higher concentrations were discarded from the experimental design because of the cell shape alterations observed in the absence of H_2_O_2_ (data not shown), not allowing to measure SO_4_^=^ uptake. On the other hand, lower concentrations were not used as ineffective. provoked, they. After 30 min incubation at 25°C, 300 μM H_2_O_2_ was added to either GSH- or curcumin-containing tube. Such treated samples were incubated for further 30 min at 25°C, before re-suspension in SO_4_^=^ medium. SO_4_^=^ uptake was then measured as described for control conditions and erythrocytes checked under a light microscope at 5 min and 90 min of incubation in SO_4_^=^ medium. Damaged erythrocytes were quantified by cell count *per* microscopic field at the end of incubation.

iii) Resealed ghosts of erythrocytes, once obtained, were re-suspended to 3% hematocrit in 35 ml isotonic medium plus 300 μM H_2_O_2_. After 30 min incubation at 25°C, samples were centrifuged to remove supernatant and resealed ghosts re-suspended to the same hematocrit in SO_4_^=^ medium. SO_4_^=^ uptake was then measured as described for control conditions.

### MDA assay

To evaluate the oxidation status of membrane, in both intact erythrocytes and resealed ghosts, treated or not with H_2_O_2_, TBARS (thiobarbituric acid reactive species) levels, resulting from reaction between TBA (thiobarbituric acid) and MDA (malondialdehyde), the end product of the lipid peroxidation process, were measured. One molecule of MDA and two molecules of TBA make up a chromogen-condensing product, formed in acidic environment, spectrophotometrically read at 532 nm [29]. In detail, treated or untreated intact erythrocytes or, alternatively resealed ghosts (10% hematocrit), were centrifuged (2700 g, 5 min) and lysed by freezing/thawing procedure in presence of 200 μM GSH. Each sample was vigorously shaken and stored at -20°C overnight. After freezing/thawing cycles, 200 μl lysed sample were treated with 0.1% v/v TBA (500 μl), freshly prepared in 1 N HCl solution. Samples were then boiled at 95°C for 1 hour, cooled in ice and centrifuged (Eppendorf, 4°C, 10000g, 15 min). The supernatant, collected from each sample, was spectrophotometrically read at 532 nm. MDA levels were measured by comparing the absorbance with a standard curve previously obtained from known amounts of MDA (Sigma, Italy) and converted to μM.

### Isolation of Band 3 and -SH groups estimation

-SH groups estimation was performed on both intact erythrocytes and resealed ghosts (untreated or treated with, alternatively, 300 μM H_2_O_2_ or 300 μM H_2_O_2_ plus antioxidants) after Band 3 protein isolation [30].

Intact erythrocytes or, alternatively, resealed ghosts, after washing with isotonic medium (10% hematocrit), were lysed by cold hypotonic buffer (2.5 mM NaH_2_PO_4_, 5 mM HEPES). After 10 min stirring at 0°C, hemoglobin and intracellular content were eliminated by repeated centrifugations (Beckman J2-21, 4°C, 17000 g, 20 min). The process, for intact erythrocytes, was repeated with the same hypotonic buffer to discard hemoglobin. One volume of membranes (from both lysed erythrocytes and lysed resealed ghosts) was then incubated with nine volumes of 0.1 M NaOH for 30 min at 0°C in presence of 200 μM DTT (dithiothreitol) and 20 μg/ml PMSF (Phenylmethylsulfonil fluoride). After incubation, samples were centrifuged (4°C, 35000 g, 45 min). The pellet, containing Band 3 protein, was washed thrice with 5 mM sodium phosphate (pH 8.0) and used for -SH groups determination. For this purpose, pellet (200 μl) was solubilized by incubating 300 μl of 20% v/v SDS (Sodium dodecyl sulphate) reagent in 3 ml of 100 mM sodium phosphate (pH 8.0), for 30 min at 37°C. Samples were further incubated with 100 μl of 10 mM DTNB (5,5'-dithiobis-(2-nitrobenzoic acid) in 100 mM sodium phosphate (pH 8.0), for 20 min at 37°C. DTNB reacts specifically with thiol groups resulting in a highly coloured yellow anion. NEM (N-ethilmaleimide, 2 mM) was used as a positive control to obtain complete oxidation of –SH groups [23]. Levels of -SH groups in the suspension were measured by spectrophotometry at 412 nm, using the molar extinction coefficient 13,600 [30], and % decrease of –SH groups with respect to untreated erythrocytes was considered.

### Reagents

All chemicals were purchased from Sigma (Milan, Italy). H_2_O_2_ was freshly prepared and diluted from 30% w/w stock solution. DIDS and GSH were dissolved in distilled water and diluted from 10 mM stock solutions. NEM was dissolved in ethanol and diluted from 1 M stock solution. Curcumin (Curcumin (1,7-bis(4-hydroxy-3-methoxyphenyl)-1E,6E-heptadiene-3,5-dione, MW = 368.4, “*pro analysis”* purity grade, Sigma, code C7727 (Milan, Italy) was kindly provided by Prof. S. Cuzzocrea (University of Messina, Italy) and dissolved in 0.5% v/v DMSO, as stock solution. Solvents (ethanol and DMSO) were preventively tested upon erythrocytes to exclude any damage.

### Experimental data and statistics

All data are expressed as arithmetic means ± S.E.M. (standard error of the mean) for statistical analysis. GraphPad Prism software (version 5.00 for Windows; GraphPad software, San Diego, CA) was used. Statistically significant differences between means were tested by paired Student’s *t* test or one-way analysis of variance (ANOVA), followed by Bonferroni's *post hoc* test and were assumed significant at p<0.05 (*p<0.05, **<0.01, ***<0.001); *N* represents the number of independent experiments. Statistical analysis, reported in both text and figure legends, refers to rate constant values for SO_4_^=^ uptake, unless differently stated.

## Results

### SO_4_^=^ uptake measurement in intact erythrocytes

#### Control conditions

**Fig 1:**
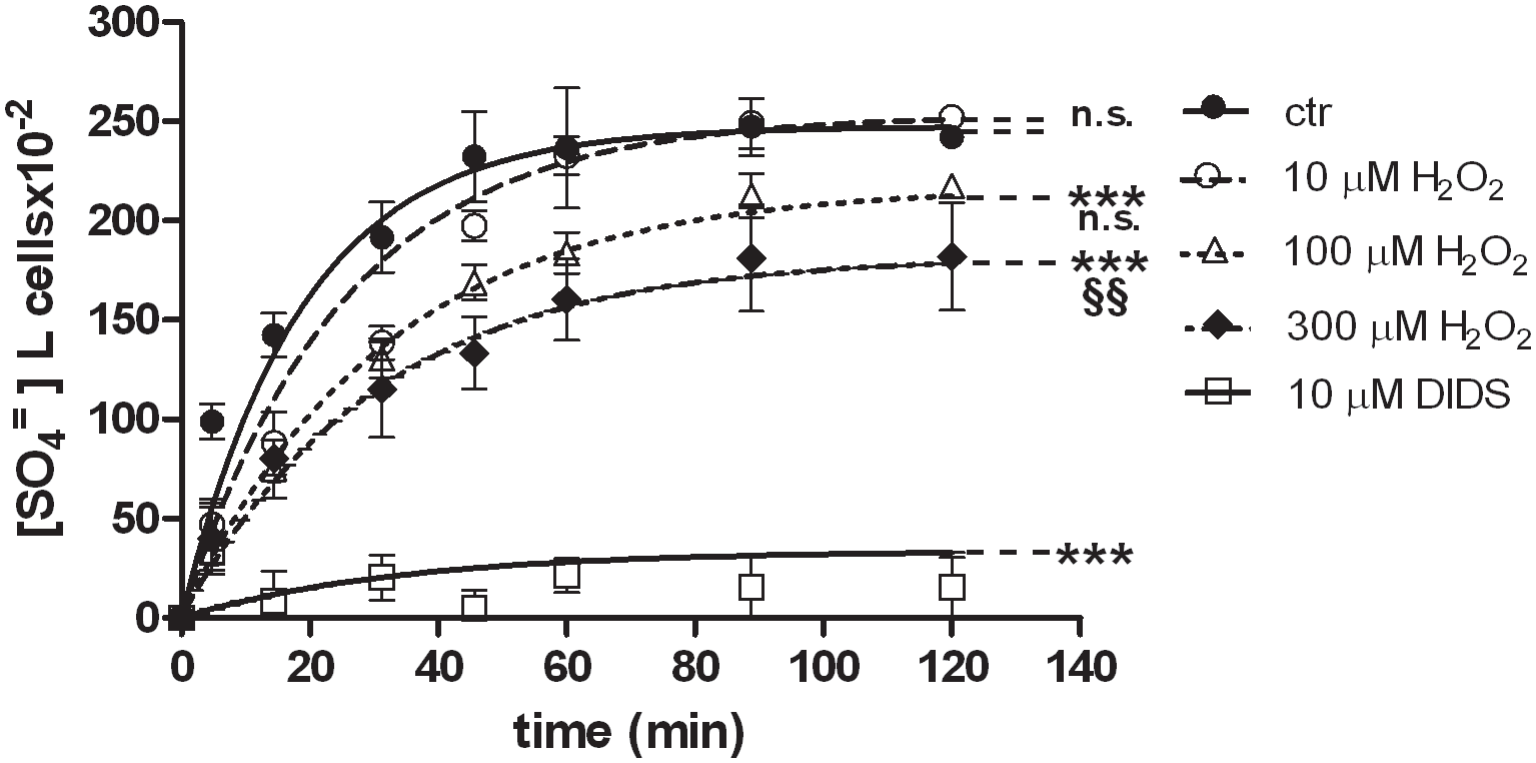
SO_4_^=^ uptake in human erythrocytes under H_2_O_2_ treatment. Time course of SO_4_^=^ uptake in human erythrocytes measured in control conditions (untreated erythrocytes) or treated with 10 μM DIDS, applied at the beginning of incubation in SO_4_^=^ medium. Points represent the mean ± SEM from separate experiments (see Table 1), where ***p<0.001 significant *versus* control and ^§§^p<0.01 significant *versus* 100 μM H_2_O_2_, as determined by one way ANOVA followed by Bonferroni's *post hoc* test, by comparing all values of theoretical curves, at all time points.

**Fig 2:**
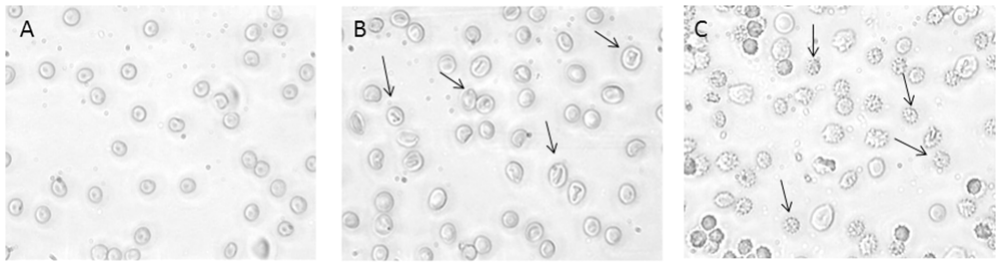
Erythrocytes morphology under H_2_O_2_ treatment. Light microscope observations (400x magnification) of: **A**) Untreated erythrocytes; **B**) 300 μM H_2_O_2_ treated erythrocytes at 5 min of SO_4_^=^ medium incubation; **C**) 300 μM H_2_O_2_ treated erythrocytes at 90 min of SO_4_^=^ medium incubation. Arrows indicate significant morphological changes in both B) and C) compared to the control (untreated) erythrocytes A).

Fig 1 describes the time course for SO_4_^=^ uptake in both treated and untreated erythrocytes. Treatment with 10 μM DIDS, applied at the beginning of incubation in SO_4_^=^ medium, completely blocked SO_4_^=^ uptake and, hence, considered as a positive control. SO_4_^=^ transport in control erythrocytes (untreated) increased steeply at the initial stage and reached equilibrium within 30 min (Fig 1), exhibiting a rate constant of 0.053±0.001 (Table 1). Both SO_4_^=^ concentrations at each time point and rate constant for SO_4_^=^ uptake in control conditions were significantly different with respect to SO_4_^=^ concentrations and rate constant of DIDS-treated cells (Fig 1, Table 1, ***p<0.001). Erythrocytes incubated in SO_4_^=^ medium did not exhibit any morphological change, as shown in Fig 2A.

**Table 1:** Rate constant for SO_4_^=^ uptake in treated and untreated erythrocytes.

Rate constant (min^-1^)		Time (min)	% decrease *vs* control	*N*
control	0.053±0.001	18	0	5
10 μM DIDS	0.029±0.0015 ***	33	45	3
10 μM H_2_O_2_	0.039±0.005 ^n. s.^	25	24	3
100 μ M H_2_O_2_	0.031±0.002 ***, ^n. s.^	31	40	4
300 μ M H_2_O_2_	0.032±0.001 ***,^§§^	30	40	6
2 mM GSH + 300 μ M H_2_O_2_	0.042±0.005 ***.^¥¥^	24	20	4
10 μM curcumin + 300 μ M H_2_O_2_	0.048±0.001 **,#	20	10	4

Rate constant (min^-1^) of SO_4_^=^ uptake in human erythrocytes measured in control conditions (untreated erythrocytes) or treated with either 10 μ M DIDS applied at the beginning of incubation in SO_4_^=^ medium or H_2_O_2_ at different concentrations (10-100-300 μ M) or 300 μ M H_2_O_2_ preceded by antioxidant (2 mM GSH or 10 μ M curcumin). Data are presented as means ± SEM from separate experiments (see *N* column), where:

***p<0.001 significant *versus* control or

**p<0.01 significant *versus* control;

^§§^p<0.01 significant *versus* 100 μ M H_2_O_2_; n.s. not significantly different *versus* 100 μ M H_2_O_2_ or 10 μ M H_2_O_2_ *versus* control;

^¥¥^ p<0.01 significant *versus* 300 μ M H_2_O_2_ and

^#^ p<0.05 *versus* 300 μ M H_2_O_2_, as determined by one way ANOVA followed by Bonferroni's *post hoc* test.

#### H_2_O_2_ treatment

As depicted in Fig 1, SO_4_^=^ uptake in erythrocytes exposed to 10 μM H_2_O_2_ did not significantly differ from SO_4_^=^ uptake in untreated cells. Exposure to 100 μM H_2_O_2_ significantly reduced SO_4_^=^ uptake when compared to untreated erythrocytes (Fig 1, ***p<0.001), while not significantly different with respect to what measured in 10 μM H_2_O_2_-treated erythrocytes (Fig 1). With regard to 300 μM H_2_O_2_ treatment, a significant SO_4_^=^ uptake inhibition was observed when compared to both control (Fig 1, ***p<0.001) and 100 μM H_2_O_2_-treated erythrocytes (Fig 1, ^§§^p<0.01). The rate constants for SO_4_^=^ uptake, accounting for this inhibition, are reported in Table 1. Concentration of SO_4_^=^ incorporated by the cells in both control and 10 μM H_2_O_2_-treated cells was significantly higher (p<0.001) than that one measured in 100 μM H_2_O_2_, 300 μM H_2_O_2_and DIDS treatment, respectively (Fig 1).

Fig 2 shows significant morphological changes in erythrocytes treated with 300 μM H_2_O_2_ at both 5 min (Fig 2B) and 90 min (Fig 2C) of SO_4_^=^ medium incubation, with respect to untreated cells (Fig 2A). Lower H_2_O_2_ concentrations (both 10 and 100 μM) did not elicit any morphological change (data not shown). In control conditions (Fig 2 A), 1% damaged erythrocytes was observed, while, at 90 min of 300 μM H_2_O_2_ treatment, this percentage raised to 98% (Fig 2C).

On this basis, 300 μM H_2_O_2_ was used for further protocols on both intact erythrocytes and hemoglobin-free resealed ghosts of erythrocytes.

**Fig 3:**
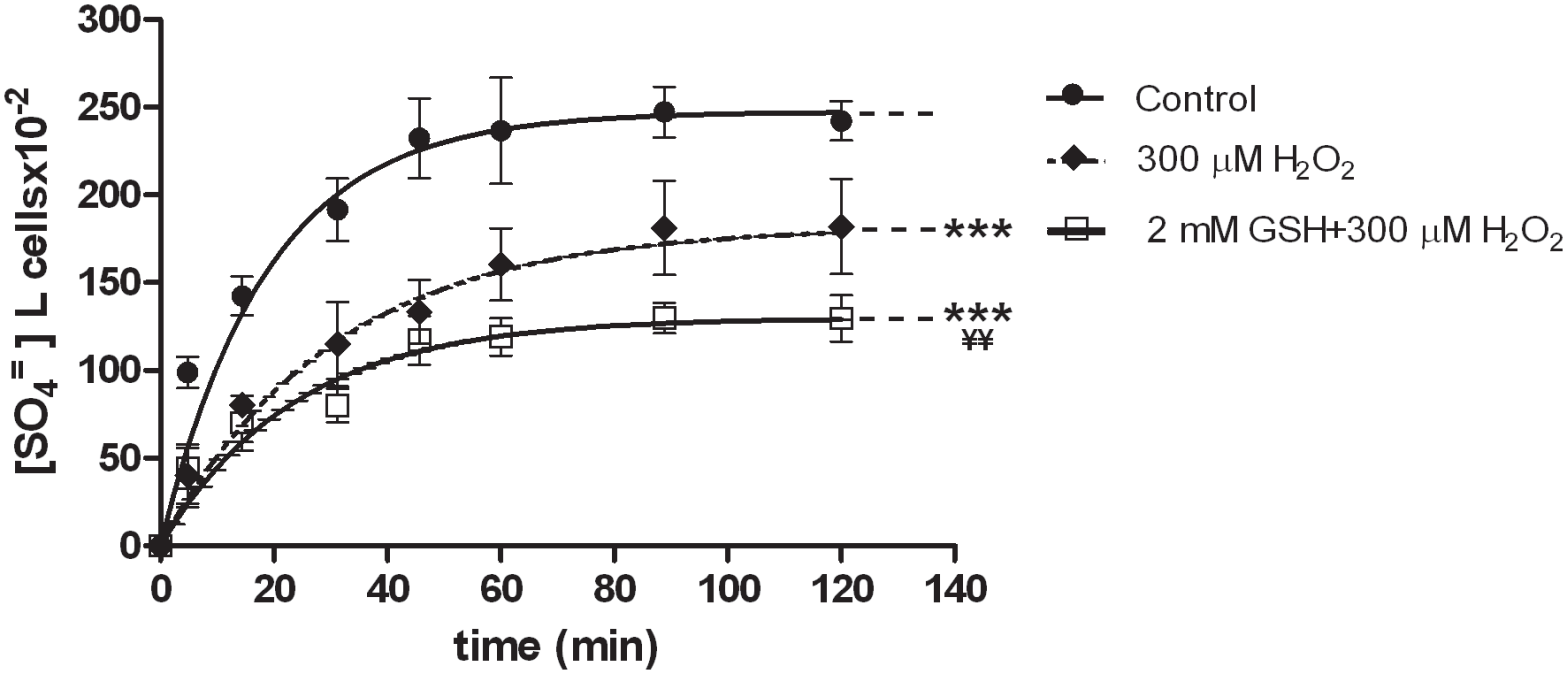
SO_4_^=^ uptake in human erythrocytes under H_2_O_2_ plus GSH treatment. Time course of SO_4_^=^ uptake in human erythrocytes measured in control conditions (untreated erythrocytes) or treated with 300 μM H_2_O_2_, or treated with 2 mM GSH and then with 300 μM H_2_O_2_. Points represent the mean ± SEM from separated experiments (see Table 1), where ***p<0.001 significant *versus* control and ^¥¥^p<0.01 significant *versus* 300 μM H_2_O_2_, as determined by one way ANOVA followed by Bonferroni's *post hoc* test, by comparing all values of theoretical curves, at all time points.

Erythrocytes, treated at first with 2 mM GSH and then with 300 μM H_2_O_2_ (Fig 3), exhibited a rate constant for SO_4_^=^ uptake significantly higher (0.042±0.005, Table 1, ^¥¥^p<0.01) than what observed in 300 μM H_2_O_2_-treated erythrocytes (0.032±0.001, Table 1), albeit significantly lower than in untreated erythrocytes (Fig 3, ***p<0.001; 0.053±0.001, Table 1).

**Fig 4:**
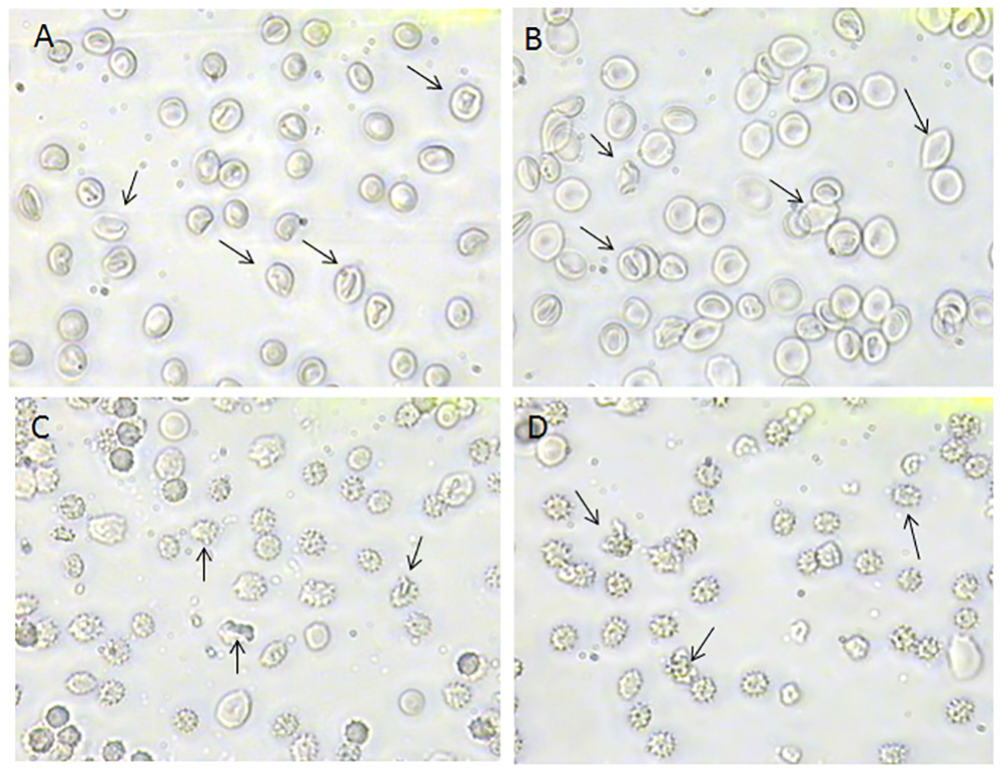
**Erythrocytes morphology under H**_**2**_**O**_**2**_ **plus GSH treatment** Light microscope observations (400x magnification) of: **A)** 300 μM H_2_O_2_ treated erythrocytes, observed after 5 min incubation in SO_4_^=^ medium and **B**) erythrocytes treated with 2 mM GSH and then with 300 μM H_2_O_2_, observed after 5 min incubation in SO_4_^=^ medium; **C)** 300 μM H_2_O_2_ treated erythrocytes, observed after 90 min incubation in SO_4_^=^ medium and **D)** erythrocytes treated with 2 mM GSH and then with 300 μM H_2_O_2_ observed after 90 min incubation in SO_4_^=^ medium. Arrows indicate that morphological changes after H_2_O_2_ treatment are not inhibited by GSH.

Fig 4 shows that morphological changes induced by 300 μM H_2_O_2_ were impaired by 2 mM GSH neither after 5 min nor 90 min of incubation in SO_4_^=^ medium, being the percentage of damaged cells unchanged after GSH plus H_2_O_2_ treatment.

**Fig 5:**
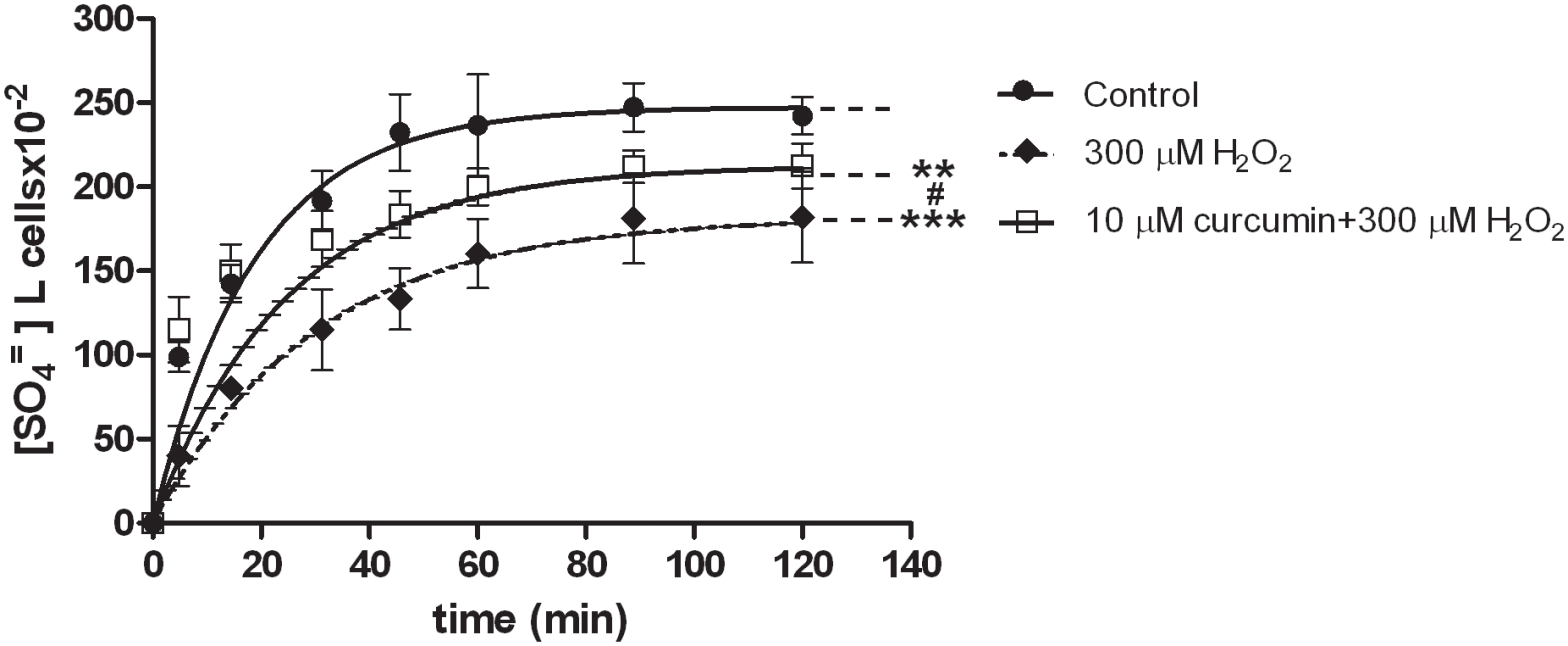
SO_4_^=^ uptake in human erythrocytes under H_2_O_2_ plus curcumin treatment. Time course of SO_4_^=^ uptake in human erythrocytes measured in control conditions (untreated erythrocytes) or treated with 300 μM H_2_O_2_ preceded or not by 10 μM curcumin application. Points represent the mean ± SEM from separate experiments (see Table 1), where ***p<0.001 significant *versus* control or **p<0.01 significant *versus* control and ^#^p<0.05 significant *versus* 300 μM H_2_O_2_, as determined by one way ANOVA followed by Bonferroni's *post hoc* test, by comparing all values of theoretical curves, at all time points.

With regard to curcumin, treatment of erythrocytes with this natural antioxidant (10 μM), followed by exposure to 300 μM H_2_O_2_ (Fig 5), impaired the H_2_O_2_-induced inhibition of SO_4_^=^ uptake, as shown by the rate constant (0.048±0.001, Table 1) significantly higher than that one measured in H_2_O_2_-treated erythrocytes (0.032±0.001, ^#^p<0.05), while significantly lower than that one observed in untreated erythrocytes (0.053±0.001, **p<0.001).

**Fig 6:**
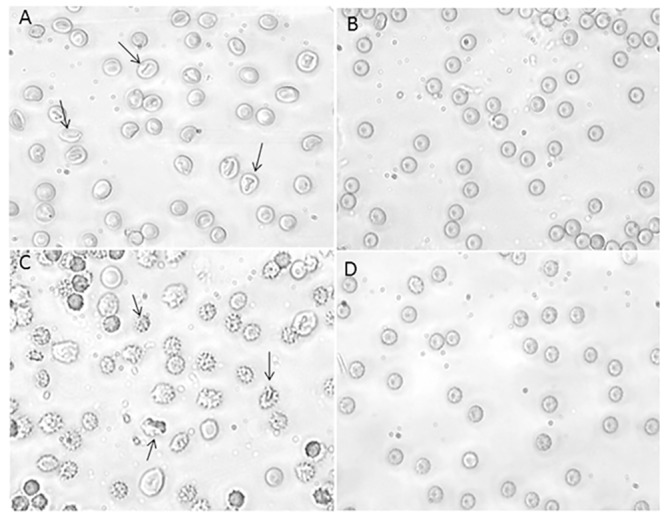
Erythrocytes morphology under H_2_O_2_ plus curcumin treatment. Light microscope observations (400x magnification) of: **A)** 300 μM H_2_O_2_ treated erythrocytes, observed after 5 min incubation in SO_4_^=^ medium and **B**) erythrocytes treated with 10 μM curcumin followed by 300 μM H_2_O_2_, observed after 5 min incubation in SO_4_^=^ medium; **C)** 300 μM H_2_O_2_ treated erythrocytes, observed after 90 min incubation in SO_4_^=^ medium and **D)** erythrocytes treated with 10 μM curcumin followed by 300 μM H_2_O_2_, observed after 90 min incubation in SO_4_^=^ medium. Arrows indicate morphological changes after H_2_O_2_ treatment, attenuated by curcumin treatment.

Fig 6 shows that morphological changes induced by 300 μM H_2_O_2_ treatment were prevented by 10 μM curcumin, at both 5 min and 90 min incubation in SO_4_^=^ medium. Percentage of damaged cells after 10 μM curcumin plus 300 μM H_2_O_2_ treatment (Fig 6D) was significantly lower (3%) than that measured after exposure to 300 μM H_2_O_2_ (98%, Fig 6C), while comparable to what measured in untreated erythrocytes (1%, Fig 2A).

### MDA assay and –SH groups estimation in erythrocytes

**Fig 7:**
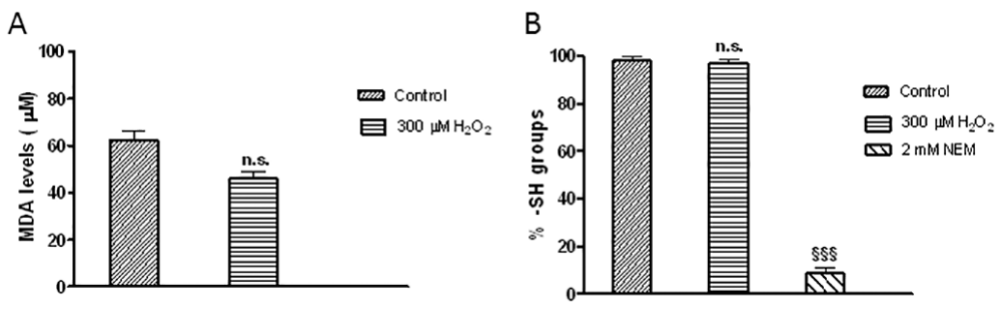
MDA levels and –SH groups estimation under H_2_O_2_ treatment. A) MDA levels observed in control (untreated erythrocytes) and in erythrocytes treated with 300 μM H_2_O_2_. Data are presented as means ± SEM from at least 3 experiments, where n.s. is not significantly different *versus* control, as determined by *t-*Student test. B) Percentage of –SH groups measured in control (untreated erythrocytes), in erythrocytes treated with either 300 μM H_2_O_2_ or 2 mM NEM. Bars represent the mean ± SEM from at least 3 experiments, where n.s. is not significant *versus* control, ^§§§^ p<0.001 significant *versus* control and 300 μM H_2_O_2_-treated erythrocytes, as determined by one way ANOVA followed by Bonferroni's *post hoc* test.

To estimate the oxidative damage induced by H_2_O_2_, MDA assay was performed. Treatment of erythrocytes with 300 μM H_2_O_2_ revealed MDA levels comparable to those observed in untreated cells (Fig 7A, n.s.). No MDA test was further carried out on erythrocytes treated with the antioxidant compounds chosen for the experimental design (GSH and curcumin).

As shown in Fig 7B, –SH groups levels measured in 300 μM H_2_O_2_-treated erythrocytes were comparable to those measured in untreated cells (Fig 7B, n.s.). These data were significantly different with respect to –SH groups levels measured in erythrocytes treated with 2 mM NEM, known as an oxidant compound and here assumed as a positive control (Fig 7B, ^§§§^ p<0.001 *versus* control and 300 μM H_2_O_2_-treated erythrocytes).

### SO_4_^=^ uptake measurement in hemoglobin-free resealed ghosts of erythrocytes

**Fig 8:**
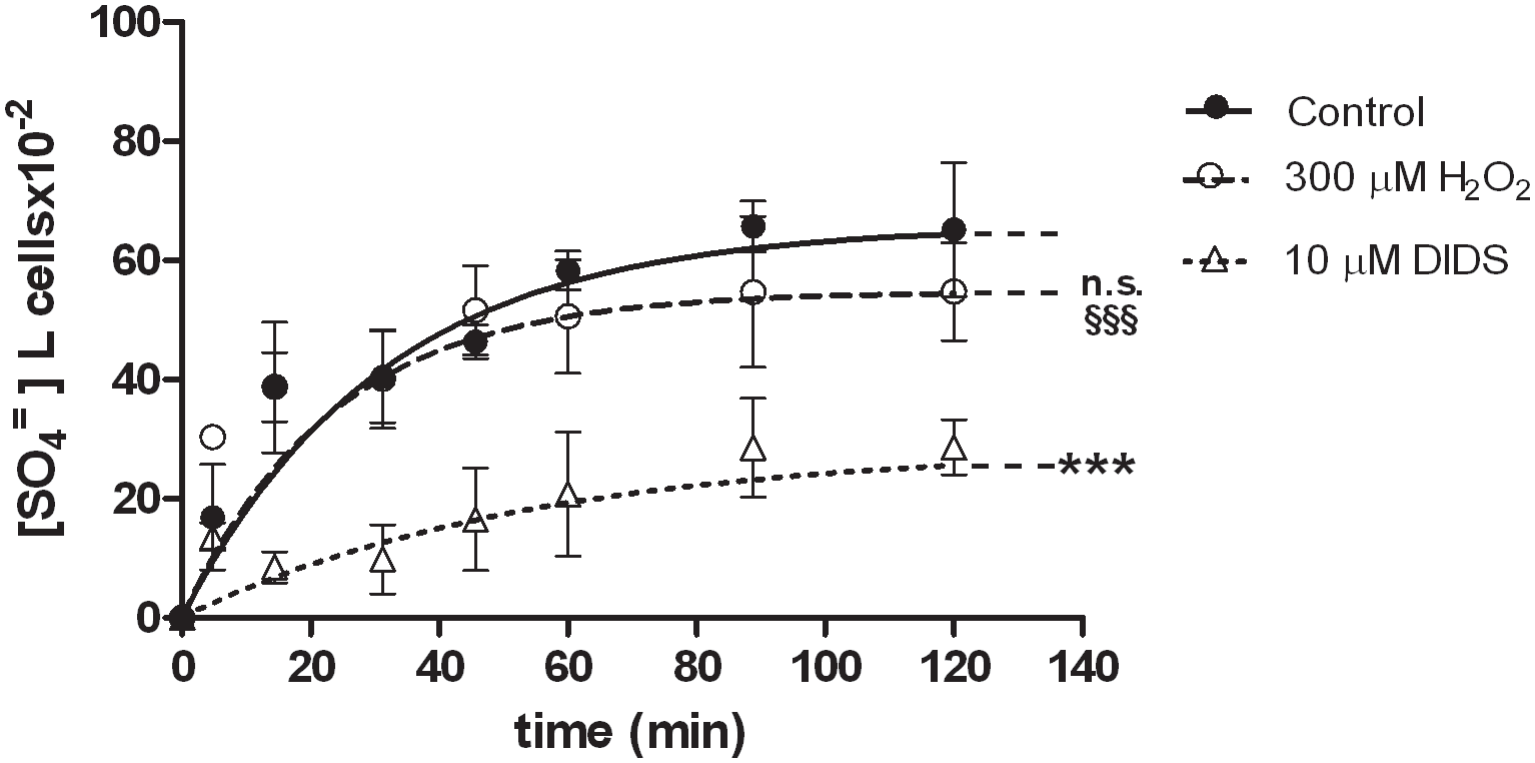
SO_4_^=^ uptake in human hemoglobin-free resealed ghosts of erythrocytes under H_2_O_2_ treatment. Time course of SO_4_^=^ uptake in hemoglobin-free resealed ghosts of erythrocytes measured in control conditions (untreated) or treated with either 300 μμM H_2_O_2_, or 10 μ M DIDS applied at the beginning of the incubation in SO_4_^=^ medium. Points represent the mean ± SEM from separate experiments (see Table 2), where ***p<0.001 significant *versus* control or ^§§§^p<0.001 significant *versus* 10 μ M DIDS and n.s. not significant *versus* control, as determined by one way ANOVA followed by Bonferroni's *post hoc* test, by comparing all values of theoretical curves, at all time points.

Fig 8 describes the time course for SO_4_^=^ uptake in untreated resealed ghosts of erythrocytes. As a positive control, 10 μM DIDS was applied at the beginning of the incubation in SO_4_^=^ medium. The rate constant for SO_4_^=^ uptake in untreated resealed ghosts was significantly higher (0.063±0.001, Table 2) than that observed in presence of DIDS (0.018±0.002 ***p<0.001). This significant difference accounted for SO_4_^=^ uptake efficiency after resealing procedures. After exposure to 300 μM H_2_O_2_, SO_4_^=^ uptake was unchanged with respect to untreated erythrocytes (Fig 8, n.s.), while significantly higher (0.042±0.005, ^§§§^ p<0.001) than in DIDS-treated cells. The rate constants for SO_4_^=^ uptake in these experimental conditions are reported in Table 2.

**Table 2:** Rate constant for SO_4_^=^ uptake in treated and untreated resealed ghosts of erythrocytes.

Rate constant (min^-1^)		Time (min)	% decrease *vs* control	*N*
control	0.063±0.001	16	0	5
10 μ M DIDS	0.018±0.002***	55	72	5
300 μ M H_2_O_2_	0.042±0.005 ^§§§^, ^n.s.^	24	34	5

Rate constant (min^-1^) of SO_4_^=^ uptake in resealed ghosts of erythrocytes measured in control conditions (untreated ghosts) or treated with either or 10 μ M DIDS applied at the beginning of the incubation in SO_4_^=^ medium or 300 μ M H_2_O_2_ Data are presented as means ± SEM of at least 5 experiments, where:

***p<0.001 significant *versus* control;

^§§§^ p<0.001 significant *versus* 10 μ M DIDS and n.s. not significant *versus* control, as determined by one way ANOVA followed by Bonferroni's *post hoc* test.

### MDA assay and –SH groups estimation in resealed ghosts

In 300 μ M H_2_O_2_-treated resealed ghosts, MDA levels, as well as –SH groups quantity, were not significantly different with respect to those measured in untreated ghosts (data not shown). Also in this case, no further protocols with H_2_O_2_ treatment combined with antioxidants was used.

## Discussion

Hydrogen peroxide (H_2_O_2_) is produced by O_2_^.-^ dismutation, inducing oxidative modifications in oxyhemoglobin [31] and generating superoxide anion radical (O_2_^.-^), the main source of ROS in erythrocytes. It has been shown that the exposure of oxyhemoglobin to H_2_O_2_ leads to increased concentration of methemoglobin, lipid peroxidation and spectrin-hemoglobin complexes [2,14]. Nevertheless, the effect of H_2_O_2_ on Band 3 protein, the most abundant protein in human erythrocytes [5], which is linked to proteins underlying erythrocytes membrane (e.g. ankyrin, adducin, protein 4.1, glycophorin) [6], is still not completely clarified.

H_2_O_2_, being a cellular metabolite, may function as a signal in the regulation of its cellular levels and, owing to the variety of its effects, the study of its actions on cells, namely erythrocytes, may become quite difficult,. Moreover, that “H_2_O_2_ signaling” may be involved in both non beneficial effects, due to too low or high H_2_O_2_ levels, and possible cell adaptation is also worthy of note ([32] and ref therein). Nevertheless, this interesting issue still remains an open question.

The aim of the present study was to better verify, in human erythrocytes, the effect of H_2_O_2_ on Band 3 protein, whose function can be monitored by measuring the efficiency in SO_4_^=^ uptake [12].

Our results show that cell shape of erythrocytes exposed for 30 min to 300 μ M H_2_O_2_ was, as expected, altered, accordingly to what already shown by Snyder and co-authors [2], who described echinocytes formation under H_2_O_2_ treatment. Furthermore, here we show a significant reduction in rate constant for SO_4_^=^ uptake under μ M H_2_O_2_ treatment, in a dose dependent manner. To possibly explain at which level this alteration occurred, oxidative effects induced by 30 min exposure to 300 μ M H_2_O_2_ were estimated by two separate methods: the first one, MDA assay, to verify the involvement of lipoperoxidation events, and the second one, -SH group measurement, to verify whether the oxidative damage was inflicted at level of membrane proteins.

Both MDA assay and -SH groups measurement revealed neither lipoperoxidation events nor –SH groups oxidation, when compared to control conditions. Hence, based on MDA levels detected in 300 μ M H_2_O_2_-treated erythrocytes, we could exclude that lipoperoxidation was responsible for rate constant reduction. This result is interesting, if compared with what shown by Mendanha and co-authors [14], observing lipid peroxidation, revealed by MDA production, in erythrocytes exposed to 300 μ M H_2_O_2_ for 3 h, a longer time than that one chosen for our experiments. In addition, the same authors demonstrated lipid peroxidation for H_2_O_2_ concentrations from 300 to 1500 μ M.

Hence, the present investigation would add one more element to what reported by Mendanha and co-authors [14], that is oxidative stress induced by a short time exposure to H_2_O_2_, whose effects are not detectable by commonly used methods, including MDA assay, affects erythrocyte function and may be revealed by monitoring SO_4_^=^ uptake through Band 3 protein. Therefore, this technique seems to be a more sensitive tool to evaluate the effects of oxidative stress on human erythrocytes function.

As far as –SH groups are concerned, we demonstrated that their levels after 300 μ M H_2_O_2_ treatment are unchanged if compared to control conditions. This observation is in line with findings from other authors [2,14], demonstrating, by means of electron spin resonance (EPR) spectroscopy, that the amount of accessible –SH groups gradually increases with H_2_O_2_ concentration, up to 300 μ M. What is more, Mendhana and co-workers [14] stated that, at H_2_O_2_ concentrations higher than 300 μ M, a decrease of –SH groups occurs, due to lipoperoxidation events causing –SH groups oxidation, as said above. So, according to these authors [14], we can suggest that in the H_2_O_2_-concentration range from 50 to 300 μM, proteins, putatively Hb molecules, bind to the membrane during oxidative stress and contribute additional -SH groups. At any rate, the reduction in rate constant for SO_4_^=^ uptake observed in H_2_O_2_-treated erythrocytes, along with cell shape modifications, can be explained with the higher amount of free –SH groups in the membrane, correlating with degree of rigidity.

That Band 3 protein may be a target of oxidative stress has been already reviewed by Lutz and Bogdanova ([11] and ref therein), reporting about clusters of this protein, associated to oxidized and denatured Hb.

As a further step supported by the hypothesis that the intracellular content of erythrocytes is involved in the response to oxidative stress induced by 30 min exposure to 300 μM H_2_O_2_, we measured SO_4_^=^ transport in H_2_O_2_-treated hemoglobin free resealed ghosts, consisting in re-constituted erythrocytes membranes deprived of intracellular content.

Resealed ghosts preparation is a validated method for drug delivery [33], for producing circulating blood analyte biosensor after entrapping fluorescence-dyes [34], and, closer to our purpose, a method to study Band 3 protein function (monitored by SO_4_^=^ uptake measurement) and its cross linking with cytoplasmic proteins [13]. The use of resealed ghosts has been recently revealed as a promising technique to study Band 3 protein capacity to carry molecules, other than those currently known [21].

Coming back to our experiments, the exposure of ghosts for 30 min to 300 μM H_2_O_2_ did not inhibit the rate constant for SO_4_^=^ uptake with respect to untreated ghosts, thus suggesting that the first target of H_2_O_2_ seems not to be the membrane, but the intracellular content, most likely Hb, which cross links with integral membrane protein, according to findings from other authors [11]. This link has been already shown, and, what is more, spectrin-Hb complex formation in human erythrocytes membrane after H_2_O_2_ treatment has been also demonstrated ([11] and ref therein), along with morphological changes and decreased cell deformability [2].

Moreover, in line with what described by Ivanov and collaborators [35] on erythrocytes exposed to an acidic medium, we may suggest that H_2_O_2_, permeating the membrane, may interfere with Hb, thus generating oxidative products, chemically reactive and capable to diffusing from cytosol to extracellular medium, thus inducing oxidative damage on membranes. In our case, H_2_O_2_, acting from the inside of the erythrocytes, would alter proteins conformation, resulting in more accessible –SH groups in the membrane. This effect would possibly obscure the oxidative damage inflicted by products deriving from Hb oxidation.

In an attempt to protect Band 3 protein from H_2_O_2_-induced oxidative stress, both GSH (2 mM) and curcumin (10 μM), have been used as antioxidant compounds. With regard to GSH experiments, the H_2_O_2_-induced reduction in rate constant for SO_4_^=^ uptake was recovered, though not completely restored, by pre-treatment of erythrocytes with GSH, whereas cell shape alterations, due to exposure to H_2_O_2_, were not impaired. Hence, we may suggest that GSH, in these experimental conditions, is not completely effective in protecting erythrocytes from oxidative damage, not preserving cell shape, which is indeed critical for erythrocytes function.

It has been already shown that GSH is not able to cross the plasma membrane, so that the intracellular GSH is the only source for erythrocytes [11,36]. This can explain why pre-treatment with GSH fails in protecting H_2_O_2_-induced cell shape alterations, detected in our experiments. Moving from this consideration, curcumin, a natural antioxidant permeating cell membrane and already known for its beneficial properties [19,37,38], has been chosen to better focus on SO_4_^=^ transport under H_2_O_2_-induced oxidative conditions in human erythrocytes.

Pre-treatment of erythrocytes with curcumin, significantly prevented both H_2_O_2_-induced cell shape alterations and reduction in rate constant for SO_4_^=^ uptake. Therefore, we may hypothesize that, in the present experimental conditions, this natural antioxidant seems to be more effective in protecting both anatomical and functional properties of erythrocytes, when exposed to oxidative stressors.

This result could be explained with the antioxidant properties of flavonoids. Some flavonoids may incorporate into the hydrophobic membrane bilayer, thus reducing its fluidity and stability [39,40], which results in a more difficult diffusion of free radicals (deriving from the cytosolic environment) and, in turn, in a more effective antioxidant power of these natural compounds. As a matter of fact, that curcumin protects SO_4_^=^ transport in human erythrocytes exposed to stressors in the external medium, like acid pH, has been recently demonstrated [23], supporting thus the hypothesis of beneficial effects of natural antioxidants.

Here we show similar effects, by proving that not only cell shape but also the rate constant for SO_4_^=^ uptake of Band 3 protein is protected by curcumin, when erythrocytes are exposed to H_2_O_2_ induced-oxidative stress. This is in line with what recently shown by Yang and co-authors [41], reporting that curcuminoids supplementation may prevent membrane dysfunction of human erythrocytes due to hyperglycemia–induced oxidative conditions.

The beneficial action of curcumin on Band 3 protein function may also open the way to further considerations, taking into account the variety of oxidative stress sources affecting human tissues, including the above mentioned hyperglycemia [41]and even the administration of radiographic contrast media, a procedure which has become very common to date. In this latter regard, Iopromide administration [42] during coronary angiography in patients with coronary artery disease has been shown to induce Band 3 protein clustering, dissociation of spectrin from Band 3 protein, loss of homogeneity of the spectrin network, echinocytes formation, and, in turn, hypo-oxygenation of the tissue, as similarly observed in the myocardium of pigs [43]. Echinocytes formation has been already described in erythrocytes [2] exposed to H_2_O_2_.

On this basis and supported by the present results, we may underline the adjuvant role of natural antioxidants assumed with food, such as curcumin, in protecting Band 3 function from external stressors, such as increase in ROS levels, which correlate with high glucose blood levels, aging, physical exercise, use of drugs and their metabolites, neurodegenerative diseases and chronic alcohol consumption [32,37,41,44,45,46]. In this latter regard, increased blood viscosity, along with impaired erythrocyte flexibility and increased fragility, have been reported as a consequence of alcohol-induced oxidation [47].

## Conclusions

Our results further confirm that, in human erythrocytes, the link between Band 3 protein and intracellular content is crucial for maintaining normal shape and, in turn, oxygen exchange efficiency; demonstrate that Band 3 protein function, monitored by SO_4_^=^ uptake measurement, is affected by low concentrated H_2_O_2_, adding more information about the effect of oxidative stress at structural level, as already assessed by other authors [2], as well as at functional level, namely correlated to Band 3 protein; a short-time exposure to low concentrations of H_2_O_2_ induces oxidative damage not detectable by MDA assay or –SH group estimation and is sufficient to alter Band 3 protein efficiency, estimated by SO_4_^=^ uptake measurement; natural antioxidants like curcumin are effective in protecting erythrocytes from this damage; Band 3 protein function assessment through SO_4_^=^ uptake measurement can be confirmed as a tool to monitor oxidative stress effect on erythrocytes.

These findings open the way to further investigations, specifically addressed to evaluate, at level of erythrocytes function, the consequences of oxidative conditions possibly provoked by strenuous physical exercise, alcohol consumption, drugs administration or neurodegenerative diseases, which have nowadays become very common. Aged erythrocytes could be also considered for future study about the impact of aging on their function, monitored through SO_4_^=^ transport measurement, related to hemoglobin modification [11,48]. The putative adaptation of erythrocytes, known as pre-conditioning response to a more or less prolonged oxidative stress [49], would be also a noteworthy issue.

## Supporting Information

S1 Text Letter signed by Dr. P. Romano, Ospedale Maggiore (Modica, RG, Italy), stating he is authorized to handle human blood samples, in accordance to the local Ethics Committee. (PDF)
