# Supplementary material for: H2O2-Induced Oxidative Stress Affects SO4= Transport in Human Erythrocytes
Source: PLoS One. 2016 Jan 8;11(1):e0146485. doi: 10.1371/journal.pone.0146485 (PMC4712827; doi:10.1371/journal.pone.0146485)

Modica, Ragusa 15 Maggio 2015

A chi di competenza

Il sottoscritto dott. Pietro Romano, dirigente medico presso l'Unità Operativa di Patologia Clinica dell'Ospedale Maggiore di Modica (Ragusa, Italia), Azienda Provinciale Sanitaria Ragusa,

dichiara

di essere autorizzato al prelievo di campioni di sangue umano, in accordo alle linee guida del comitato etico locale.

In fede

Pietro Romano, MD
Dirigente medico U.O. Patologia Clinica
Ospedale Maggiore Modica (Ragusa)
ASP Ragusa Italy
Tel +39.328 2631367

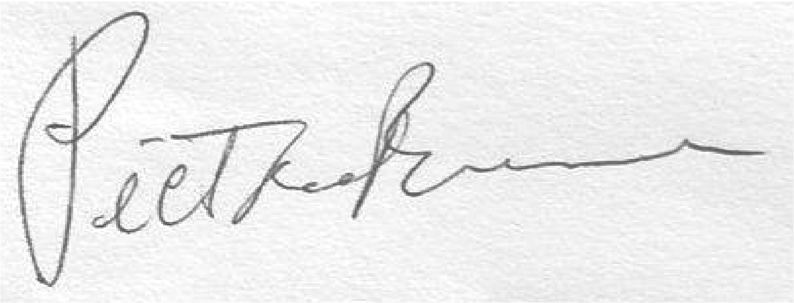

Supplement: S1 Text — (PDF) [file pone.0146485.s001.pdf]
